# Self-assembly and immunogenicity of virus-like particles from goose astrovirus 1 structural protein following proteolytic maturation in a baculovirus system

**DOI:** 10.1016/j.psj.2025.106037

**Published:** 2025-10-30

**Authors:** Anping Wang, Zhi Wu, Li Liu, Qingkang Zhou, Yuting Cheng, Wenfeng Jia, Huipeng Lu, Jun Xie, Shanyuan Zhu

**Affiliations:** Jiangsu Agri-Animal Husbandry Vocational College, Jiangsu Key Laboratory for High-Tech Research and Development of Veterinary Biopharmaceuticals, Taizhou 225300, PR China

**Keywords:** Goose astrovirus 1, Virus-like particles, Baculovirus/insect cell system, Characterization, Immunogenicity

## Abstract

Goose astrovirus 1 (**GAstV-1**) is a significant pathogen responsible for enteritis and visceral gout in goslings, yet no commercial vaccine is currently available. The difficulty in efficiently culturing the virus in vitro has hindered the development of traditional vaccines. Virus-like particles (**VLPs**), which lack genetic material and mimic native viral conformation, represent a promising vaccine strategy. In this study, the full-length ORF2 structural protein of GAstV-1 was efficiently expressed using a baculovirus/insect cell system. Maximum expression was observed at an MOI of 5 and 5 days post-infection in both extracellular and intracellular fractions. Western blot analysis indicated that ORF2 undergoes proteolytic cleavage, producing mature proteins of 40/43 kDa core and 25/27 kDa spike fragments. Transmission electron microscopy of ultrathin sections revealed abundant VLPs associated with cytoplasmic membranes, and negatively stained purified VLPs showed typical particles of approximately 30 nm accompanied by 10 nm ring-like structures. Immunization of goslings with as little as 5 μg VLPs induced high levels of specific antibodies and a mixed Th1/Th2 immune response. Following challenge, viral shedding was significantly suppressed in immunized groups, with protection comparable to an inactivated virus vaccine. This work establishes a robust platform for GAstV-1 VLP production, provides new insights into proteolytic processing of the viral structural protein, and demonstrates the strong immunogenicity and protective efficacy of VLPs, supporting their potential as a subunit vaccine against GAstV-1.

## Introduction

Goose astrovirus 1 (**GAstV-1**) has emerged as a significant pathogen severely threatening the goose industry in recent years. It primarily causes an acute infectious disease characterized by enteritis in goslings under three weeks of age and is frequently associated with co-infections with goose astrovirus 2 (**GAstV-2**), leading to gout in goslings characterized by urate deposition in joints and viscera (Zhou, et al., 2024; Chen, et al., 2025a; Wang, et al., 2025a). The mortality rate can reach up to 50 %, resulting in substantial economic losses to the global waterfowl industry.

GAstV-1 belongs to the family *Astroviridae* and possesses a single-stranded, positive-sense RNA genome of approximately 7.2 kb in length that contains three open reading frames (**ORF1a, ORF1b**, and **ORF2**). ORF2 encodes the sole structural protein, with a molecular weight of approximately 87-90 kDa. From the N- to the C-terminus, this protein comprises the S, P1, P2, and acidic domains (Arias and DuBois, 2017). Studies on human astrovirus 1 (**HAstV-1**) have shown that the initial ORF2 product (**VP90**) is first cleaved intracellularly by caspases to remove the C-terminal acidic domain and part of the N-terminal sequence, generating an approximately 70 kDa intermediate (**VP70**). Subsequently, extracellular cleavage by trypsin-like proteases further processes VP70 into the mature capsid proteins VP34 (34 kDa) and VP27 (27 kDa), ultimately leading to the formation of mature virions (Méndez, et al., 2002; Méndez, et al., 2004). VP34 contains the S and P1 domains, which together form the core of the viral capsid, while VP27 comprises the P2 domain, forming the surface spikes responsible for receptor binding, viral entry, and the presentation of major neutralizing epitopes (York, et al., 2015; Toh, et al., 2016).

At present, no commercial vaccine against GAstV-1 is available, and disease prevention relies primarily on biosecurity measures (Ren, et al., 2025). The development of traditional inactivated and live attenuated vaccines is limited due to the difficulty of efficiently propagating the virus in cell culture systems. Consequently, GAstV-1 amplification mainly relies on goose embryos. However, the limited availability of specific-pathogen-free (**SPF**) goose embryos and generally low virus titers severely restrict the large-scale production and application of traditional vaccines (Wang, et al., 2021; Wang, et al., 2025a). Therefore, the development of safe and effective novel vaccines is urgently needed.

Virus-like particles (**VLPs**) are non-infectious, hollow particles composed solely of viral structural proteins that retain the native virion conformation. They lack viral genetic material, thus offering excellent safety profiles. Simultaneously, VLPs retain the native conformational epitopes of the virus, enabling them to effectively elicit both humoral and cellular immune responses in the host. Consequently, VLPs have become a crucial strategy in novel vaccine development. Currently, VLP-based vaccines (e.g., for human papillomavirus and hepatitis B virus) have been successfully marketed and have demonstrated remarkable efficacy (Bachmann, et al., 2025). However, research on astrovirus VLPs remains scarce, with only two published studies focusing on HAstV-1 VLPs (Dalton, et al., 2003; Caballero, et al., 2004). Whether the GAstV-1 ORF2 structural protein can be efficiently expressed in eukaryotic expression systems, correctly self-assembled into VLPs, and confer protective immunity has not yet been investigated. In this study, we employed the Bac-to-Bac baculovirus expression system to achieve successful expression of the full-length GAstV-1 ORF2 protein. We optimized expression conditions, confirmed VLP formation by transmission electron microscopy, and evaluated immunogenicity and protective efficacy in goslings. The findings provide both theoretical insights and technical support for the development of GAstV-1 subunit vaccines.

## Materials and methods

### Cells, virus, and antibodies

*Spodoptera frugiperda* (**Sf9**, Invitrogen, USA) cells were cultured in Sf-900™ II SFM serum-free medium (Gibco, USA) in shake flasks at 27 °C and 150 r/min. The GAstV-1 TZ03 strain (GenBank accession No. MW353015) was isolated and preserved by Jiangsu Key Laboratory for High-Tech Research and Development of Veterinary Biopharmaceuticals (Wang, et al., 2021). Virus stocks were prepared by inoculating 9-day-old SPF goose embryos via the allantoic cavity. The recombinant GAstV-1 ORF2 protein was expressed in *Escherichia coli* (***E.coli***) and purified by Ni-NTA affinity according to the published method (Wang, et al., 2025b). The mouse monoclonal antibody (**mAb**) A5A1, directed against the P1 domain of GAstV-1 ORF2, was prepared in our laboratory and has been verified to specifically bind native virus (Wang, et al., 2025b).

### Construction of recombinant baculovirus expressing GAstV-1 ORF2

The recombinant baculovirus was generated using the Bac-to-Bac baculovirus/insect cell system. Using RNA extracted from the GAstV-1 TZ03 strain as template, the full-length ORF2 ORF was amplified by RT-PCR with ORF2-specific primers (ORF2-F: 5′-TGCGGATCCATGGCCGACAAGGTCACTGTC-3′; ORF2-R: 5′-GCACTCGAGTTAATCAAACTCTTGTCCGCC-3′). The PCR product was digested with *BamH* I and *Xho* I and directionally cloned into the similarly digested baculovirus transfer vector pFastBac1, yielding the recombinant plasmid pFastBac-GAstV1-ORF2. The purified recombinant plasmid was transformed into DH10Bac competent cells. Following Tn7-mediated transposition and blue-white screening, white colonies were selected and verified by PCR using M13 universal primers, resulting in the recombinant bacmid Bacmid-GAstV1-ORF2 containing the target ORF2 sequence. The purified bacmid was then transfected into Sf9 cells in the logarithmic growth phase using Cellfectin II Reagent (Invitrogen). After incubation at 27 °C under serum-free conditions for 5 h, fresh medium was added, and the culture was continued for 5 days. When typical cytopathic effects (cell swelling and detachment) appeared, the culture supernatant was collected as the P1 stock of recombinant virus, designated rBac-GAstV1-ORF2. The P1 virus was amplified by infecting Sf9 cells at a multiplicity of infection (**MOI**) of 0.1 for two successive rounds to obtain a stable, wild-type-free P3 virus stock, which was stored at 4 °C protected from light. The titer of the P3 virus was determined by plaque assay.

### Preparation of mouse polyclonal antibody against the GAstV-1 ORF2-P2 domain

A 690 bp fragment corresponding to nt 6177-6866 of the GAstV-1 TZ03 genome, which encodes the P2 domain of ORF2, was amplified by high-fidelity PCR using gene-specific primers P2-F (5′-TAAGAAGGAGATATACATATGCAGAACCTACCACTCATCTATG-3′) and P2-R (5′-TCAGTGGTGGTGGTGGTGGTGAGTTTTGAGGGAACACTTGAAGG-3′). The PCR product was cloned into the prokaryotic expression vector pET-30a via homologous recombination using the ClonExpress II One Step Cloning Kit (Vazyme, Nanjing, China), resulting in the recombinant plasmid pET-ORF2-P2. The construct was transformed into *E.coli* BL21(DE3) competent cells, and the expression of the His-tagged ORF2-P2 fusion protein was induced with 0.5 mmol/L IPTG. The recombinant protein was purified by Ni²⁺-NTA affinity chromatography, and its purity was confirmed to be over 90% by SDS-PAGE. Six-week-old BALB/c mice were immunized intraperitoneally with 50 μg of purified protein emulsified in an equal volume of Freund’s adjuvant. Booster immunizations were administered every 14 days for a total of three immunizations. Serum was collected 7 days after the final immunization to obtain mouse polyclonal antibodies (**pAb**) against ORF2-P2.

### Analysis of recombinant protein expression

Sf9 cells in the logarithmic growth phase were suspended at a density of 2 × 10⁶ cells/mL in 100 mL medium and infected with the rBac-GAstV1-ORF2 at MOI of 1, 5, and 10, followed by incubation at 27 °C with shaking at 150 r/min. Starting at 72 hours post-infection (**hpi**), 10 mL of cell culture was aseptically collected every 24 hours. The samples were centrifuged at 3,000 × g for 15 min at 4 °C to separate the culture supernatant from the cell pellet. The supernatant was concentrated by ultracentrifugation at 200,000 × g for 2 h at 4 °C, and the resulting pellet was resuspended in 1 mL of phosphate-buffered saline (**PBS**). The cell pellet was resuspended in 1 mL of PBS and subjected to three freeze-thaw cycles to lyse the cells. After centrifugation at 15,000 × g for 10 min at 4°C, the supernatant (soluble fraction) and the precipitate (insoluble fraction, resuspended in 1 mL PBS) were collected separately. All collected fractions were analyzed by Western blot (**WB**) to evaluate the expression level and distribution of the recombinant protein under different MOIs, time points, and subcellular components.

### Indirect immunofluorescence assay (IFA)

Sf9 cells were seeded into a 24-well plate at a density of 6 × 10⁵ cells per well and allowed to adhere for 30 min at 27 °C. Monolayers were then infected with rBac-GAstV1-ORF2 at an MOI of 5. After 2 h of incubation at 27 °C, fresh medium was added, and the infection continued for 48 h. The infected cells were gently washed three times with PBS, fixed with 4 % paraformaldehyde for 20 min at room temperature (**RT**), and permeabilized with 0.5 % Triton X-100 for 10 min at RT. Non-specific binding sites were blocked with 10 % fetal bovine serum (**FBS**) in PBS for 2 h at RT. Cells were then incubated overnight at 4°C with anti-ORF2 mAb A5A1 diluted 1:1,000 in blocking buffer, followed by three PBS washes. Subsequently, fluorescein isothiocyanate (**FITC**)-conjugated goat anti-mouse IgG (Jackson ImmunoResearch, USA) diluted 1:200 was applied for 1 h at RT in the dark. After thorough washing with PBS, images were acquired using a fluorescence inverted microscope.

### Western blot (WB)

Protein samples were mixed with 4 × reducing loading buffer and denatured at 95 °C for 5 min. After separation on 12 % SDS-PAGE gels, proteins were transferred onto a 0.22 μm polyvinylidene fluoride membrane using a wet transfer system. The membrane was blocked with 5% skim milk in PBST for 2 h at RT, followed by incubation with either a 1:1,000 dilution of the anti-ORF2 mAb A5A1 or a 1:200 dilution of the anti-ORF2-P2 pAb overnight at 4 °C. After washing three times with PBST, the membrane was incubated with a 1:10,000 dilution of horseradish peroxidase (**HRP**)-conjugated goat anti-mouse IgG (Jackson ImmunoResearch) for 1 h at RT. Following extensive washing with PBST, immunoreactive bands were visualized using an enhanced chemiluminescence detection kit.

### Preparation and purification of GAstV-1 virus-like particles (VLPs)

Sf9 cells in the logarithmic growth phase were seeded at a density of 2 × 10⁶ cells/mL in a 250 mL Erlenmeyer flask containing 50 mL of culture medium and infected with rBac-GAstV1-ORF2 at an MOI of 5. Culture was incubated at 27 °C with shaking at 150 r/min for 5 days. Cell cultures were harvested and centrifuged at 2,000 × g for 10 min at 4 °C to separate the supernatant from the cell pellet. The cell pellet was resuspended in 5 mL of PBS and subjected to three freeze-thaw cycles for cell lysis, and then centrifuged at 15,000 × g for 10 min at 27°C to obtain the cell lysate supernatant. The culture supernatant was further concentrated by ultracentrifugation at 200,000 × g for 3 h at 4 °C, and the resulting pellet was resuspended in 5 mL of PBS.

Both the cell lysate supernatant and the concentrated culture supernatant were layered onto the top of a discontinuous sucrose density gradient (20%, 40% and 60% w/v) and subjected to ultracentrifugation at 200,000 × g for 3 h at 4 °C. Visible bands were collected sequentially from the bottom to the top of the gradient, diluted in PBS, and pelleted again at 200,000 × g for 3 h at 4°C. The final pellets were resuspended in 1 mL PBS, aliquoted, and stored at -80°C until further use. WB with mAb A5A1 confirmed the identity of the VLP-containing fractions, and protein concentrations were determined by the BCA assay. GAstV-1 TZ03 allantoic fluid was processed in parallel for concentration, purification and quantification following the same procedure.

### Electron microscopy

For intracellular visualization, Sf9 cells were infected with rBac-GAstV1-ORF2 at an MOI of 5 and harvested at 72 h post-infection. Cells were fixed with 2.5 % glutaraldehyde in 0.1 M PBS (pH 7.4) for 2 h at 4°C, washed three times with the same buffer, and post-fixed with 1 % osmium tetroxide for 1 h at 4°C. After dehydration through an ethanol gradient, the samples were embedded in Epon-812 resin. Ultrathin sections (∼70 nm) were cut on an ultramicrotome, double-stained with 2 % uranyl acetate and lead citrate, and examined with a transmission electron microscope (**TEM**, UC6, Leica, Germany) to determine the morphology and subcellular localization of VLPs. For negative staining, purified GAstV-1 VLPs were applied to 300-mesh copper grids for 1 min, washed briefly with distilled water, and stained with 2 % phosphotungstic acid (pH 7.4) for 1 min. After air-drying at RT, grids were examined and imaged using a Tecnai 12 TEM (FEI, USA).

### Animal experiments

All animal procedures were conducted in accordance with the Guidelines for the Care and Use of Laboratory Animals and were approved by the Institutional Animal Care Committee of Jiangsu Agri-animal Husbandry Vocational College (Approval ID: JSAHVC-2025-16). A total of thirty 3-day-old healthy Taizhou goslings, confirmed negative for both GAstV-1 and its antibodies, were randomly divided into five groups (n = 6 each). Groups 1-3 received 5, 10, or 20 µg of purified GAstV-1 VLPs, respectively; group 4 received 10 µg of β-propiolactone-inactivated purified GAstV-1 allantoic fluid; and group 5 received an equal volume of PBS as a negative control. All antigens were emulsified 1:1 (v/v) with MONTANIDE ISA 206 adjuvant (Seppic, Shanghai, China). Prime and identical booster immunizations were administered intramuscularly into the leg on days 0 and 14. Blood samples were collected weekly via the jugular vein, and serum was separated and stored at -80°C until analysis. GAstV-1-specific antibody levels were detected by indirect ELISA. Concentrations of interleukin-4 (**IL-4**) and interferon-γ (**IFN-γ**) in serum were measured using commercial goose-specific ELISA kits (Milbio, Shanghai, China) according to the manufacturer’s instructions. On day 14 after the booster immunization, all goslings were challenged orally with 0.3 mL of GAstV-1 TZ03 strain (0.3 × 10^3.25^ TCID50/goose). Clinical symptoms were observed daily post-challenge. Cloacal swabs were collected every 3 days up to 15 days post-challenge (**dpc**). Viral shedding was quantified by quantitative real-time PCR (**qPCR**) targeting the GAstV-1 ORF1b gene.

### Indirect ELISA

Flat-bottom 96-well microplates were coated overnight at 4°C with 100 µL per well of purified GAstV-1 ORF2 recombinant protein (1.0 µg/mL) in 50 mM carbonate buffer (pH 9.6). After five washes with PBST (0.05 % Tween-20 in PBS), each well was blocked with 100 μL of PBS containing 5% skim milk for 2 h at 37 °C, followed by another five washes with PBST. Next, 100 μL of test serum diluted in blocking buffer was added to each well and incubated for 1 h at 37 °C. After washing, 100 μL of HRP-conjugated goat anti-duck IgG antibody (KPL, USA) diluted at 1:1000 was added and incubated for 1 h at 37 °C. The reaction was developed using TMB (3,3′,5,5′-tetramethylbenzidine) substrate for 15 min in the dark and stopped with 2 M H₂SO₄. The absorbance was measured at 450 nm using a microplate reader (Bio-Rad).

### Viral load detection

Total RNA was extracted from cloacal swab samples using the MagicPure Up Viral DNA/RNA Kit (TransGen, Beijing, China) according to the manufacturer’s instructions. The extracted RNA was used as a template to synthesize cDNA using the TransScript One-Step RT-PCR SuperMix (TransGen). GAstV-1 genomic copies were quantified using a previously established qPCR method developed in our laboratory (Wang, et al., 2022), results are expressed as log₁₀ copies/μL.

### Statistical analysis

All experimental data were statistically analyzed with GraphPad Prism version 9.5.0 (GraphPad Software Inc., USA) and are presented as mean ± standard deviation (**SD**). A P-value of less than 0.05 was considered statistically significant.

## RESULTS

### Expression of GAstV-1 ORF2 structural protein in insect cells

**Fig. 1:**
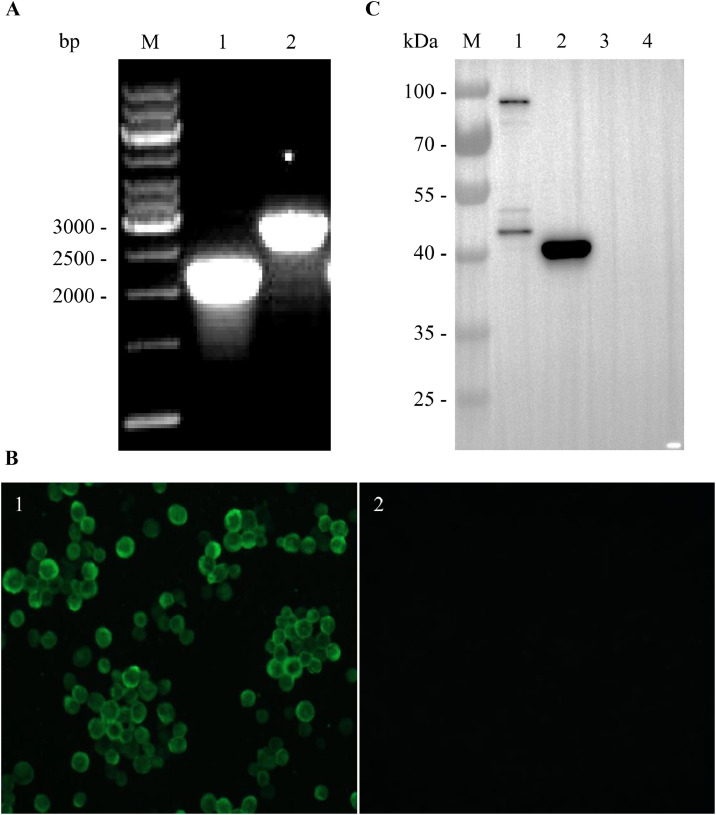
Expression of goose astrovirus 1 (**GAstV-1**) ORF2 structural protein in Sf9 insect cells. (A) PCR identification of the recombinant bacmid rBacmid-GAstV1-ORF2. M, DNA molecular weight marker; lane 1: PCR product using ORF2-F as the forward primer and M13-R as the reverse primer; lane 2: PCR product using M13-F as the forward primer and ORF2-R as the reverse primer. (B) Indirect immunofluorescence assay (**IFA**) of ORF2 expression. Sf9 cells were infected with recombinant baculovirus rBac-GAstV1-ORF2 at a multiplicity of infection (**MOI**) of 5. After 2 days, cells were fixed and probed with the ORF2-P1-specific monoclonal antibody (**mAb**) A5A1. (C) Western blot (**WB**) analysis of ORF2 expression. Sf9 cells were infected with rBac-GAstV1-ORF2 at an MOI of 5. At 3 days post-infection, both culture supernatant and cell pellets were collected and analyzed by WB using mAb A5A1. M, pre-stained protein molecular weight marker; lane 1: culture supernatant of rBac-GAstV1-ORF2-infected Sf9 cells; lane 2: cell pellet of rBac-GAstV1-ORF2-infected Sf9 cells; lane 3: culture supernatant of mock-infected Sf9 cells; lane 4: cell pellet of mock-infected Sf9 cells. A prominent 40 kDa band in the cell pellet (lane 2) and additional bands of approximately 90, 75, 50 and 43 kDa in the culture supernatant (lane 1) were observed, indicating differential proteolytic processing between intracellular and extracellular fractions.

To express the GAstV-1 ORF2 structural protein, the ORF2 ORF was cloned into the baculovirus transfer vector pFastBac1, resulting in the recombinant plasmid pFastBac-GAstV1-ORF2. Transformation of DH10Bac competent cells with this construct, followed by Tn7-mediated site-specific transposition and blue/white screening, produced the recombinant bacmid rBacmid-GAstV1-ORF2 (Fig. 1A). Transfection of purified bacmid DNA into Sf9 cells generated the recombinant baculovirus rBac-GAstV1-ORF2. After three rounds of amplification, the viral titer reached approximately 2.8 × 10⁸ PFU/mL. Sf9 cells were infected with rBac-GAstV1-ORF2 at an MOI of 5. Expression of the ORF2 protein was analyzed by IFA and WB using the anti-GAstV-1 ORF2 mAb A5A1. IFA results showed specific green fluorescence in infected Sf9 cells, whereas no signal was detected in mock-infected controls (Fig. 1B). WB analysis showed a prominent 40 kDa band in cell lysates and additional bands of approximately 90, 75, 50 and 43 kDa in the culture supernatant (Fig. 1C), indicating differential proteolytic processing of the ORF2 structural protein. No specific bands were detected in uninfected cells or their culture medium. Collectively, these results confirm the successful expression of the GAstV-1 ORF2 structural protein in insect cells.

### Optimization of expression conditions for GAstV-1 ORF2 structural protein

**Fig. 2:**
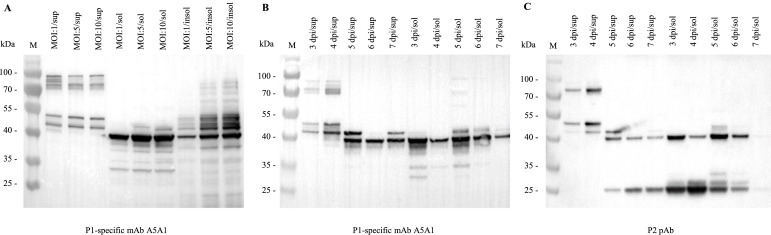
Optimization of expression conditions for goose astrovirus 1 (**GAstV-1**) ORF2 structural protein expression in Sf9 insect cells. (A) Expression of the ORF2 structural protein at different multiplicities of infection (**MOIs**). Sf9 cells were infected with recombinant baculovirus rBac-GAstV1-ORF2 at MOIs of 1, 5, and 10. At 3 days post-infection, the culture supernatant (**sup**), soluble cell lysate (**sol**), and insoluble cell lysate pellet (**insol**) were collected and analyzed by Western blot (**WB**) using ORF2-P1-specific monoclonal antibody (**mAb**) A5A1 as the primary antibody. (B, C) Expression of the ORF2 structural protein at different time points and cellular fractions. Sf9 cells were infected with rBac-GAstV1-ORF2 at an MOI of 5. From 3 to 7 days post-infection (**dpi**), the culture supernatant (sup), soluble cell lysate (sol), and insoluble cell lysate pellet (insol) were collected every 24 hours and analyzed by WB using mAb A5A1 (B) and the ORF2-P2 mouse polyclonal antibody (**pAb**) (C) as primary antibodies, respectively.

To optimize the expression of the GAstV-1 ORF2 structural protein in Sf9 cells, rBac-GAstV1-ORF2 was used to infect cells at different MOI. Starting at 3 days post-infection (**dpi**), both the culture supernatants and cell pellets were collected at 24 h intervals. The supernatants were concentrated by ultracentrifugation, while the cell pellets were subjected to freeze-thaw cycles followed by centrifugation to separate the soluble lysates from the insoluble fractions. WB analysis using the mAb A5A1 revealed that an MOI of 5 or 10 yielded the highest ORF2 levels in the culture supernatant, soluble cell lysates and the insoluble fractions (Fig. 2A).

Using an MOI of 5, we further examined the kinetics of recombinant protein expression with either ORF2-P1 mAb A5A1 or ORF2-P2 pAb. In the culture supernatant, ORF2 was barely detectable at 3 dpi, accumulated steadily thereafter and reached maximal levels at 5 dpi (Fig. 2B). In the soluble cell lysates, the highest expression was observed between 3 and 5 dpi, followed by a gradual decline (Fig. 2C).

To delineate the processed forms of ORF2, both culture supernatant and soluble cell lysate were analyzed using both antibodies. When probed with ORF2-P1 mAb A5A1, culture supernatants exhibited multiple bands of ∼90, 75, 50, 43 and 40 kDa; by 5 dpi, the 43 and 40 kDa species became predominant. In the soluble cell lysates at 3 dpi, the 40 kDa band was most prominent, accompanied by minor bands at ∼35 and 30 kDa; with prolonged infection, the pattern simplified to the 40 kDa species (Fig. 2B). Using the ORF2-P2 pAb, the soluble cell lysates displayed the 40 kDa band together with additional 25 and 27 kDa fragments, whereas supernatants harvested at 3-4 dpi were dominated by 75, 50 and 43 kDa bands, shifting to 40 and 25 kDa bands by 5 dpi (Fig. 2C).

Collectively, GAstV-1 ORF2 was successfully expressed in both culture supernatant and intracellular fractions. Maximal yields were obtained when Sf9 cells were infected at an MOI of 5 and harvested at 5 dpi.

### Formation of GAstV-1 virus-like particles (VLPs)

**Fig. 3:**
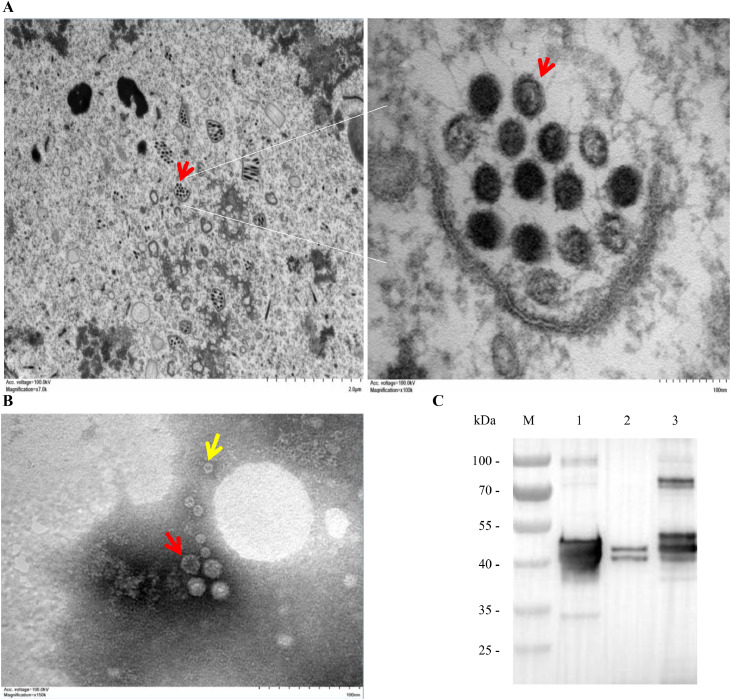
Characterization of self-assembled goose astrovirus 1 (**GAstV-1**) virus-like particles (**VLPs**) in recombinant baculovirus-infected Sf9 cells. (A) Transmission electron microscopy (**TEM**) image of ultrathin sections of Sf9 cells infected with rBac-GAstV1-ORF2 at an MOI of 5. Cells were harvested at 3 days post-infection, processed for ultrathin sectioning, and observed under TEM. (B) Negative-staining TEM image of purified GAstV-1 VLPs. VLPs were concentrated by ultracentrifugation, purified via sucrose density gradient centrifugation, negatively stained with 2% phosphotungstic acid, and observed under TEM. Red arrows indicate VLPs with a diameter of approximately 30 nm, yellow arrows indicate ring-shaped VLPs approximately 10 nm in size. Original magnification: × 150,000; scale bar: 100 nm. (C) Western blot (**WB**) analysis of purified VLPs. Sf9 cells were infected with rBac-GAstV1-ORF2 at an MOI of 5. At 5 days post-infection, culture supernatant and soluble cell lysate were collected, concentrated, purified, and analyzed by WB using ORF2-P1-specific monoclonal antibody (**mAb**) A5A1. M, pre-stained protein molecular weight marker; lane 1: VLPs purified from culture supernatant; lane 2: VLPs purified from soluble cell lysate; lane 3: purified GAstV-1 virions.

To determine whether the ORF2 protein expressed in insect cells self-assembles into VLPs, ultrathin sections of Sf9 cells infected with rBac-GAstV1-ORF2 were examined by TEM. Numerous spherical virus-like particles, morphologically and dimensionally consistent with native astroviruses, were observed within the cytoplasm, predominantly in close association with membrane structures (Fig. 3A). No such particles were detected in uninfected controls.

Following concentration of culture supernatants and soluble cell lysates by ultracentrifugation and purification through a discontinuous sucrose density gradient, negative-stain TEM revealed two distinct particle populations: particles approximately 30 nm in diameter, and smaller ring-shaped structures about 10 nm in size (Fig. 3B).

WB analysis of gradient-purified fractions, using the mAb A5A1, showed that VLPs derived from culture supernatants contained a heterogeneous population of structural proteins with molecular masses of ∼90, 75, 50, 43, 40 and 30 kDa, whereas those from the soluble cell lysates were enriched in 40 and 43 kDa species. In parallel, purified GAstV-1 virions from infected allantoic fluid displayed a comparable proteolytic pattern dominated by 90, 75, 50, 43 and 40 kDa bands (Fig. 3C). These findings demonstrate that the GAstV-1 ORF2 structural protein expressed in insect cells can spontaneously assemble into VLPs with morphology and protein composition similar to those of the wild-type virus.

### Immunogenicity of GAstV-1 VLPs

**Fig. 4:**
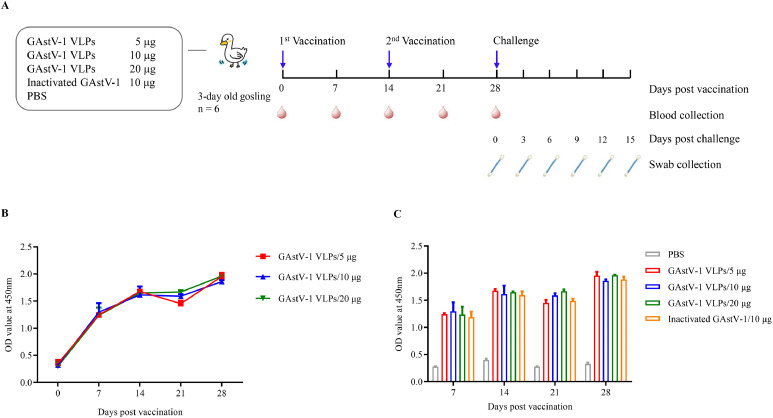
Goose astrovirus 1 (**GAstV-1**) virus-like particles (**VLPs**) induce specific antibody responses in goslings. (A) Schematic diagram of the experimental design in goslings, including group allocation, immunization schedule, challenge time, and sampling plan. (B, C) Indirect ELISA results of serum from immunized goslings. Three-day-old goslings were immunized with different doses of GAstV-1 VLPs or inactivated GAstV-1 emulsified with MONTANIDE ISA 206 adjuvant. Serum samples were collected weekly post-immunization. Specific antibody titers were detected by indirect ELISA using purified ORF2 protein as the coating antigen. Data are presented as mean ± standard deviation (**SD**), with each sample measured in triplicate. Statistical analysis was performed using GraphPad Prism version 9.5.0.

To evaluate the immunogenicity of GAstV-1 VLPs, 3-day-old goslings were intramuscularly immunized with 5, 10, or 20 μg of purified VLPs, while a positive-control group received 10 μg of β-propiolactone-inactivated GAstV-1 allantoic fluid (Fig. 4A). Indirect ELISA results showed that all VLP-immunized groups elicited high levels of specific antibodies as early as 7 days after the primary immunization, with peak responses observed at 14 days that were further elevated after the booster dose. No significant differences in antibody levels were observed among the three VLP doses (P > 0.05, Fig. 4B), and titers were statistically indistinguishable from those elicited by the inactivated virus (Fig. 4C).

**Fig. 5:**
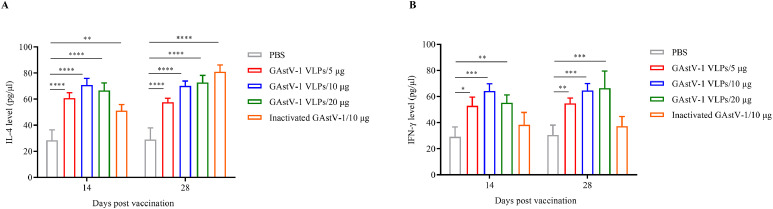
Cytokine responses induced by goose astrovirus 1 (**GAstV-1**) virus-like particles (**VLPs**) in goslings. Serum levels of interleukin-4 **(IL-4**, A) and interferon-gamma (**IFN-γ**, B) were measured by indirect ELISA at 14 and 28 days post-immunization in goslings immunized with VLPs or inactivated GAstV-1. Data are presented as mean ± standard deviation (**SD**), with each sample measured in triplicate. Statistical analysis was performed using GraphPad Prism version 9.5.0. *P < 0.05, **P < 0.01, ***P < 0.001, ****P < 0.0001.

Cytokine detection further revealed that both VLP and inactivated virus groups significantly increased IL-4 levels in the blood of goslings at 14 days after primary and booster immunizations (P < 0.01). No significant differences in IL-4 levels were observed among the VLP dose groups (Fig. 5A). In contrast, IFN-γ levels were significantly elevated only in the VLP groups (P < 0.05), while the inactivated virus group showed no difference compared to the negative control (Fig. 5B). These findings indicate that VLPs can simultaneously activate a mixed Th1/Th2 immune response.

**Fig. 6:**
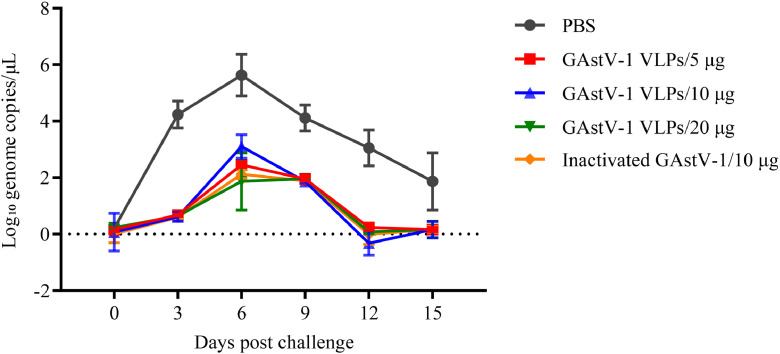
Protective efficacy of goose astrovirus 1 (**GAstV-1**) virus-like particles (**VLPs**) in a challenge model in goslings. Cloacal swabs were collected every 3 days post-challenge from goslings immunized with different antigens. Viral load was quantified by qPCR. Data are presented as mean ± standard deviation (**SD**), with each sample measured in triplicate. Statistical analysis was performed using GraphPad Prism version 9.5.0.

At 14 days after the booster immunization, all goslings were challenged orally with GAstV-1 TZ03. Throughout the observation period, no clinical symptoms or weight loss were observed in any immunized group. Quantitative PCR analysis of cloacal swabs showed that the negative control group began shedding virus at 3 dpc, reached peak shedding at 6 dpc, and continued until 15 dpc. In contrast, both VLP and inactivated virus groups exhibited only minimal viral shedding at 6 and 9 dpc, with no significant differences in shedding levels among the immunized groups (P > 0.05, Fig. 6). These results demonstrate that GAstV-1 VLPs can significantly suppress viral shedding comparable to that of inactivated virus.

## Discussion

GAstV-1 has emerged in recent years as a critical pathogen severely threatening the healthy breeding of goslings. Due to its difficulty in efficient propagation in vitro cell culture systems (Zhu and Sun, 2022; Zhai, et al., 2025), the development of traditional vaccines has faced significant challenges. Virus-like particles, which are empty particles self-assembled from structural proteins without viral genetic material, offer excellent safety and strong immunogenicity, and have become an important strategy for novel vaccine development (Kheirvari, et al., 2023). In this study, we demonstrate, for the first time, the robust expression of full-length GAstV-1 ORF2 in a baculovirus/insect cell expression system, its autonomous assembly into morphologically authentic VLPs, and the induction of protective immunity in goslings. These findings establish a scalable production platform for GAstV-1 VLPs and provide compelling evidence for their utility as a subunit vaccine candidate.

Selection of an appropriate expression platform is pivotal for successful VLP production. Commonly employed systems include prokaryotic hosts, yeast, baculovirus/insect cells, and mammalian cells. Selection among these requires comprehensive consideration of factors such as the characteristics of the target protein, the complexity of assembly, production costs, and biosafety (Bachmann, et al., 2025). The sole structural protein of astroviruses is encoded by ORF2, yet its conversion into mature virions requires sequential intracellular caspase cleavage followed by extracellular trypsin-like processing (DuBois, et al., 2013; Aguilera-Flores, et al., 2022). To date, only two studies have reported astrovirus VLPs: one using a baculovirus system for HAstV-1 and the other employing a vaccinia virus system for HAstV-2. Here, we selected the baculovirus/insect cell platform to express full-length GAstV-1 ORF2. WB analysis revealed a heterogeneous proteolytic pattern, with bands ranging from 25 to 90 kDa present in both cell lysates and culture supernatants. This profile closely parallels the HAstV-1 ORF2 expression pattern previously described (Caballero, et al., 2004), but differs markedly from the single 87 kDa band reported for HAstV-2 expressed in vaccinia virus (Dalton, et al., 2003). This discrepancy may stem from differences in post-translational processing capabilities between baculovirus and vaccinia systems, and/or from strain-specific proteolytic cleavage sites among different astroviruses (Delgado-Cunningham, et al., 2022; Ghosh, et al., 2024). The presence of multiple proteolytic products in our study indicates that the baculovirus system enables effective post-translational processing and cleavage of the GAstV-1 ORF2 protein, which is essential for the formation of correctly conformational epitopes and the assembly of VLPs.

Previous studies have shown that the HAstV structural protein ORF2 is cleaved intracellularly by caspases and extracellularly by trypsin-like proteases to yield VP34 and VP27 (Arias and DuBois, 2017). VP34 forms the core of the virion, encompassing the S and P1 domains, whereas VP27 contains the P2 domain and constitutes the surface spikes. For GAstV-1, however, detailed characterization of the structural protein has so far been limited to in silico predictions and sequence alignments, with no experimental validation available. In this study, as the infection time increased, the protein bands gradually became more uniform and mature, indicating that the ORF2 structural protein underwent a series of intracellular and extracellular hydrolysis events to eventually form the mature protein forms. WB analyses employing the P1-specific mAb A5A1 consistently identified dominant bands at approximately 40 kDa and 43 kDa, whereas the P2-directed pAb revealed bands at 25 kDa and 27 kDa. These observations suggest that the 40/43 kDa species may represent the core structural proteins of GAstV-1, while the 25/27 kDa fragments may correspond to the spike domain. Together, these data provide preliminary experimental evidence for elucidating the protein composition of GAstV-1 virions. Further validation using techniques such as mass spectrometry analysis will be necessary in future studies.

The assembly of astrovirus particles is a complex process in which membrane structures are likely to play an important role (Mendez, et al., 2007). Studies have indicated that the replication complex of HAstV anchors to the perinuclear endoplasmic reticulum membrane via a double-arginine motif at the N-terminus of its nonstructural protein (Bengert, et al., 2025). Furthermore, the membrane-bound form of the structural protein VP90 (**mVP90**) is believed to be associated with the initial steps of viral morphogenesis (Ali, et al., 2024; Xiang, et al., 2024). The assembly mechanism of avian astroviruses remains unclear. In this study, VLPs were observed in the cytoplasm of infected cells, and these particles were predominantly clustered around membrane structures. This phenomenon is consistent with observations in HAstV-infected cells (Bub, et al., 2023), suggesting that GAstV-1 assembly may similarly be associated with specific organelle membranes, much like its human counterpart. Additionally, two morphological structures were identified in the purified VLP samples: intact virus-like particles approximately 30 nm in diameter and ring-shaped structures about 10 nm in size. A similar phenomenon has been reported in studies on HAstV-1 VLPs (Caballero, et al., 2004). This report demonstrated that ORF2 expressed in insect cells could assemble into ∼38 nm VLPs and ∼16 nm ring-shaped particles, showing high antigenic similarity between the two forms. The ring-shaped structures observed alongside VLPs may represent assembly intermediates, disassembled subviral particles, or alternatively, misfolded capsid proteins. Due to technical limitations, the two morphological forms observed in this study could not be separated, purified, or analyzed in depth for their protein composition and antigenic properties. This remains an important objective for future research. Nevertheless, sucrose gradient centrifugation purification confirmed that both structures could be recognized by anti-ORF2 specific antibodies and exhibited a protein composition highly similar to that of the wild-type virus. This indicates that the GAstV-1 ORF2 protein expressed in insect cells is capable of self-assembling into VLPs with native conformational epitopes. Combining electron microscopy observations and protein analysis results, we demonstrate that the GAstV-1 ORF2 protein expressed in insect cells possesses strong self-assembly capability, forming VLPs that mimic the morphology and antigenicity of natural viral particles. These findings support the conclusion that the baculovirus/insect cell expression system is suitable for producing GAstV-1 VLPs.

The development of vaccines against GAstV remains at an exploratory stage. Recent studies, such as the bivalent vaccine based on a recombinant goose-derived Newcastle disease virus expressing the GAstV-2 capsid protein (Xu, et al., 2019) and the GAstV-2 ORF2 gene delivered by a duck enteritis virus vector (Chen, et al., 2025b), have focused exclusively on GAstV-2, leaving GAstV-1 vaccines an unmet need. The GAstV-1 VLPs prepared in this study demonstrated excellent immunogenicity. High levels of specific antibodies were detected as early as 7 days after the primary immunization in all VLP-immunized groups, and antibody titers increased further following booster immunization, reaching levels comparable to those induced by inactivated virus. In terms of cellular immunity, VLP immunization significantly induced the production of both IL-4 and IFN-γ, indicating the successful activation of a mixed Th1/Th2 immune response, which is essential for effective viral clearance and comprehensive protection. In contrast, the inactivated vaccine only elevated IL-4 levels, suggesting a bias toward a Th2 response. The induction of IFN-γ by VLPs, but not by the inactivated virus, may be attributed to the particulate nature and native conformational epitopes of VLPs, which are potentially more effective in being taken up and presented by antigen-presenting cells, thereby promoting a stronger Th1-type cellular immune response. Challenge experiments confirmed that VLP immunization significantly suppressed viral shedding in cloacal swabs. Since GAstV-1 exhibits low mortality in one-month-old goslings (An, et al., 2020; Wang, et al., 2025a), no deaths or obvious clinical symptoms were observed in the control group. Therefore, the protective efficacy of the VLPs could not be directly assessed based on clinical signs or mortality. Future studies should refine the immunization schedule (e.g., earlier vaccination) or employ a maternal immunization model to evaluate passive antibody transfer and protection of progeny, thereby providing a more complete appraisal of the VLP vaccine candidate.

## Conclusion

This study represents the first successful expression of GAstV-1 ORF2 protein using a baculovirus/insect cell system and demonstrate its ability to self-assemble into morphologically uniform VLPs, thereby providing a key technological platform for GAstV-1 vaccine development. WB profiling allowed us to propose a model in which the mature GAstV-1 virion comprises a core protein of ∼40/43 kDa and a spike protein of ∼25/27 kDa, offering the first experimental insight into the proteolytic processing of GAstV-1 structural proteins and laying the groundwork for future structural biology studies. A VLP-based vaccine induced a balanced Th1/Th2 mixed immune response and significantly suppressed viral shedding, demonstrating substantial potential for disease prevention and control. Future research should focus on refining downstream processes to reduce production costs, exploring the feasibility of developing multivalent vaccines incorporating antigens from other goose pathogens, and elucidating the molecular mechanisms underlying VLP assembly and protective immunity. These efforts will provide a novel and efficient strategy for the prevention and control of GAstV-1.

## Funding

This investigation received financial support through the Science & Technology Innovation Team Program (grant number: NSF2023TC02) administered by Jiangsu Agri-Animal Husbandry Vocational College.

## CRediT authorship contribution statement

**Anping Wang:** Conceptualization, Funding acquisition, Project administration, Writing – original draft, Writing – review & editing. **Zhi Wu:** Conceptualization, Project administration, Writing – review & editing. **Li Liu:** Data curation, Methodology, Resources. **Qingkang Zhou:** Data curation, Methodology. **Yuting Cheng:** Methodology. **Wenfeng Jia:** Methodology. **Huipeng Lu:** Software. **Jun Xie:** Validation. **Shanyuan Zhu:** Conceptualization, Funding acquisition, Project administration, Writing – review & editing.

## Disclosures

The authors declare that they have no known competing financial interests or personal relationships that could have appeared to influence the work reported in this paper.
